# Production of the compatible solute α-d-glucosylglycerol by metabolically engineered *Corynebacterium glutamicum*

**DOI:** 10.1186/s12934-018-0939-2

**Published:** 2018-06-16

**Authors:** Benjamin Roenneke, Natalie Rosenfeldt, Sami M. Derya, Jens F. Novak, Kay Marin, Reinhard Krämer, Gerd M. Seibold

**Affiliations:** 1 Institute of Biochemistry, University of Cologne, Zülpicher Str. 47, 50674 Cologne, Germany; 2 Institute of Microbiology and Biotechnology, Ulm University, Albert-Einstein Allee 11, 89081 Ulm, Germany; 3 Present Address: Gutachterbüro U. Borchardt, Hennef (Sieg), Germany; 4 Present Address: Evonik Degussa GmbH, Halle (Westphalia), Germany

**Keywords:** *Corynebacterium glutamicum*, Trehalose, Compatible solute, Glycogen, α-d-Glucosylglycerol

## Abstract

**Background:**

α-d-Glucosylglycerol (αGG) has beneficial functions as a moisturizing agent in cosmetics and potential as a health food material, and therapeutic agent. αGG serves as compatible solute in various halotolerant cyanobacteria such as *Synechocystis* sp. PCC 6803, which synthesizes αGG in a two-step reaction: The enzymatic condensation of ADP-glucose and glycerol 3-phosphate by GG-phosphate synthase (GGPS) is followed by the dephosphorylation of the intermediate by the GG-phosphate phosphatase (GGPP). The Gram-positive *Corynebacterium glutamicum*, an industrial workhorse for amino acid production, does not utilize αGG as a substrate and was therefore chosen for the development of a heterologous microbial production platform for αGG.

**Results:**

Plasmid-bound expression of *ggpS* and *ggpP* from *Synechocystis* sp. PCC 6803 enabled αGG synthesis exclusively in osmotically stressed cells of *C. glutamicum* (pEKEx2-*ggpSP*), which is probably due to the unique intrinsic control mechanism of GGPS activity in response to intracellular ion concentrations. *C. glutamicum* was then engineered to optimize precursor supply for αGG production: The precursor for αGG synthesis ADP-glucose gets metabolized by both the *glgA* encoded glycogen synthase and the *otsA* encoded trehalose-6-phosphate synthase. Upon deletion of both genes the αGG concentration in culture supernatants was increased from 0.5 mM in *C. glutamicum* (pEKEx3-*ggpSP*) to 2.9 mM in *C. glutamicum* ΔotsA IMglgA (pEKEx3-*ggpSP*). Upon nitrogen limitation, which inhibits synthesis of amino acids as compatible solutes, *C. glutamicum* ΔotsA IMglgA (pEKEx3-*ggpSP*) produced more than 10 mM αGG (about 2 g L^−1^).

**Conclusions:**

*Corynebacterium glutamicum* can be engineered as efficient platform for the production of the compatible solute αGG. Redirection of carbon flux towards αGG synthesis by elimination of the competing pathways for glycogen and trehalose synthesis as well as optimization of nitrogen supply is an efficient strategy to further optimize production of αGG.

**Electronic supplementary material:**

The online version of this article (10.1186/s12934-018-0939-2) contains supplementary material, which is available to authorized users.

## Background

Accumulation of compatible solutes is required for the acclimation of organisms to adverse environmental conditions with increasing external osmolality, particularly to salt and drought stress [1, 2]. Compatible solutes are low molecular weight compounds, which can be accumulated in the cytoplasm to molar concentrations without negatively affecting cellular functions and metabolism [3]. Additionally, these solutes are able to protect cellular macromolecules directly by stabilizing their hydration shell [4–6]. When accumulated in response to hyperosmotic stress compatible solutes increase the internal osmolality, which safeguards water uptake and constant turgor pressure [1, 7]. The accumulation of compatible solutes is achieved by either de novo synthesis or uptake. Both processes are part of the immediate response to osmotic stress and comprise the direct activation of preformed enzymes [8].

Currently many compatible solutes such as ectoine, hydroxyectoine, and trehalose are widely used as moisturizing agents in cosmetics [9, 10], stabilizing agents for enzyme preparations, and/or as xeroprotectants for the storage of biomaterials and mammalian cells [11–13]. The compatible solute α-d-glucosylglycerol (αGG) has various beneficial functions and a large potential as a health food material, therapeutic agent, and for enzyme stabilization [14, 15]. αGG serves as a compatible solute in various halotolerant cyanobacteria such as *Synechocystis* sp. PCC 6803 [16, 17]. In this organism αGG is synthesized in a two-step reaction in which the enzymatic condensation of ADP-glucose and glycerol 3-phosphate by GG-phosphate synthase (GGPS) is followed by the dephosphorylation of the intermediate by the GG-phosphate phosphatase (GGPP) [18, 19]. The mechanism for the control of αGG synthesis in *Synechocystis* sp. PCC 6803 was recently described. In the absence of salt stress, the enzyme GgpS is bound to DNA in inactive form. Upon an increase in the extracellular salt concentration, Na^+^ enters the cell. The rise in the cytoplasmic Na^+^ concentration interferes with the binding of the GgpS protein to DNA leading to the detachment of the enzyme. As a result, the enzyme is activated and catalyzes the synthesis of α-glucosylglycerol-phosphate [20]. Enzymatic syntheses using different glycosyltransferases (e.g. sucrose phosphorylase from *Leuconostoc mesenteroides*, α-glucosidase from *Halomonas* sp. strain H11, or amylosucrase from *Methylobacillus flagellates*) were developed [21–23] and are currently applied for the industrial production of αGG. These enzymatic processes depend on pure substrates [22], as impurities lead to formation of unwanted by-products or inhibition of the process. In difference the use of microbes as biocatalyst allows feedstock flexibility, as the cells can be genetically engineered to efficiently generate required precursors [24, 25]. Recently, *Synechocystis* sp. PCC 6803 was optimized for αGG production [26]. For this purpose, *ggpR* as well as *ggtC* and *ggtD* were disrupted, which encode the transcriptional repressor GgpR of *ggpS* and *ggpP* and the components of the αGG uptake system [17], respectively. The resulting *Synechocystis* sp. PCC 6803 strain produced extracellular αGG, however, it required first hyperosmotic stress for intracellular αGG synthesis via GGPS and GGPP and then a switch to hypoosmotic conditions to trigger αGG secretion via mechanosensitive channels in the culture broth [26]. Engineering of non-natively αGG synthesizing bacteria towards production of this compatible solute has hitherto not been described [15].

The Gram-positive soil bacterium *Corynebacterium glutamicum*, a well-established industrial workhorse for amino acid production [27, 28], has been genetically engineered into a suitable platform for the production of various bulk and commodity chemicals [25, 29]. Recently, *C. glutamicum* has been genetically engineered towards efficient production of the compatible solutes trehalose and ectoine [30–34]. *C. glutamicum* itself is highly osmotolerant and accumulates by synthesis or uptake large amounts of compatible solutes in response to hyperosmotic stress [35]. When cultivated in minimal medium *C. glutamicum* wild type mainly synthesizes the amino acid proline as compatible solute in response to hyperosmotic stress. When nitrogen is limiting trehalose is synthesized as compatible solute via the OtsAB and the TreYZ pathways [36, 37].

In this communication, we describe the genetic engineering of *C. glutamicum* for the production of αGG. This organism transiently accumulates in the course of cultivations with sugars as substrate large amounts of glycogen [36, 38–42]. As glycogen synthesis depends on the formation of the intermediate ADP-glucose [41], which is also the precursor for αGG synthesis, it seemed likely that *C. glutamicum* might be suited as αGG production organism. In addition, formation of the second αGG precursor glycerol-3-phosphate has been observed in *C. glutamicum* cells [43]. We overexpressed *ggpS* and *ggpP* from *Synechocystis* sp. PCC 6803 in *C. glutamicum* and thus enabled αGG synthesis in osmotically stressed cells of the derived strain. We further analyzed molecular targets to optimize supply of the αGG precursor ADP-glucose and optimize cultivation conditions. By this means we here describe for the first time a strategy for efficient microbial production of αGG.

## Methods

### Bacterial strains, media, and culture conditions

Bacteria and plasmids used in this study are listed in Table 1. *E. coli* and all pre-cultures of *C. glutamicum* were grown aerobically in TY complex medium [44] at 37 and 30 °C, respectively, as 50-mL cultures in 500-mL baffled Erlenmeyer flasks on a rotary shaker at 120 rpm. For the main cultures of *C. glutamicum*, cells of an overnight pre-culture were washed twice with 0.9% (w/v) NaCl and then inoculated into CGC minimal medium [45] containing glucose or sucrose as carbon sources as indicated in “Results” section. When appropriate, kanamycin (25 or 50 µg mL^−1^), spectinomycin (30 µg mL^−1^), carbenicillin (30 µg mL^−1^), and/or isopropyl-β-d-thiogalactopyranoside (IPTG, 250 µM) were added to the media. For growth experiments in presence of nitrogen-limitation CgC-medium without ammonia and varying amounts of urea were used as indicated in “Results” section; salt stress was induced by direct addition of NaCl to cultivation vessels to final concentrations indicated in “Results” section. Growth of *E. coli* and of *C. glutamicum* was followed by measuring the optical density at 600 nm (OD_600_). For analyses of αGG production in bioreactors growth experiments were performed aerobically at 30 °C as 1 L cultures in 1.5 L jars in a Biostat B fermentation system from Braun essentially as described before [40]. The pH was maintained at 7.0 by online measurement using a standard pH electrode (Mettler Toledo) and the addition of 4 M KOH and 15% (v/v) H_2_SO_4_. Dissolved oxygen was measured online by use of a polarimetric oxygen electrode (Mettler Toledo) and it was adjusted to 30% of saturation in a cascade by stirring at 100 to 1200 rpm and aerating with 3 L of air per minute.

**Table 1:** Strains and plasmids used in this study

Strain/plasmid	Relevant characteristics	References/source
*E. coli* strains		
DH5α	*F*^−^ *Φ80lacZ*ΔM15Δ(*lacZYA*-*argF*) *U169 phoA SupE44, hsdR17, recA1, endA1, gyrA96, thi*-*1. relA1*	[68]
BL21(DE3)	*ompT hsdS*_B_(r_B_^−^ m_B_^−^) *gal dcm* (DE3)	[69]
*C. glutamicum* strains		
WT	Wild-type strain ATCC 13032	American type culture collection
Δ*otsA*	*C. glutamicum* WT with deletion of *otsA* (cg2907)	[37]
IM*glgA*	*C. glutamicum* WT with inactivation of *glgA* (cg1268)	This work
Δ*otsA* IM*glgA*	*C. glutamicum* WT with deletion of *otsA* and inactivation of *glgA*	This work
Δ*otsA* Δ*treY* Δ*treS*	*C. glutamicum* WT with deletion of *otsA* (cg2907), *treY* (cg2323), and *treS* (cg2529)	[37]
Plasmids		
pEKEx2	Kan^r^; *C. glutamicum*/*E. coli* shuttle vector (*P*_*tac*_, *lacI*^q^; pBL1, *oriV*_*C. glutamicum*_, *oriV*_*E. coli*_)	[70]
pEKEx2-GgpP	Derived from pEKEx2, for expression of *ggpP* in *C. glutamicum*	This work
pEKEx2-GgpPS	Derived from pEKEx2, for expression of *ggpP* and *ggpS* in *C. glutamicum*	This work
pEKEx3	Spt^r^; *C. glutamicum*/*E. coli* shuttle vector (*P*_*tac*_, *lacI*^q^; pBL1, *oriV*_*C. glutamicum*_, *oriV*_*E. coli*_)	[71]
pEKEx3-GgpPS	Derived from pEKEx3, for expression of *ggpP* and *ggpS* in *C. glutamicum*	This work
pDrive	Kan^r^, Amp^r^; *E. coli* cloning vector (*lacZ*α, *orif1*, *ori*-pUC)	Qiagen, Hilden, Germany
pDrive-IMglgA	Derived from pDrive, vector carrying internal region of *glgA* (cg1268) for inactivation	This work
pASK-IBA3	Overexpression vector (STREP TagII); Amp^r^	IBA GmbH, Göttingen, Germany
pASK-IBA3-*otsA*	Derived from pASK-IBA3, for regulated expression of *otsA* (*cg2907*) of *C. glutamicum*	This work

### Analysis of product and substrate concentrations

αGG concentrations in culture supernatants were analyzed using GC as recently described [20]. In the course of fermentation experiments substrate and product (sucrose and αGG) concentrations in the culture supernatants were analyzed using a Hitachi HPLC system equipped with a L2130 gradient pump, a L2200 column oven, an L2350 auto sampler, a L2490 RI detector, and a Nucleo Sugar 810Ca (300/7.8) column plus Nucleogel Sugar 810 Ca (30/4) precolumn (Macherey & Nagel). The mobile phase was 0.005 M CaCl_2_ in water with a flow rate of 0.7 mL min^−1^, the column temperature was 60 °C and the sample volume 5 µL. Calibration was performed using a serial dilution of a solution containing 50 mM sucrose (Sigma-Aldrich) and 50 mM αGG (Bitop AG). The dry weight of *C. glutamicum* in the course of cultivations was calculated according to the OD_600_; as described recently [40].

For the analysis of intracellular αGG concentrations 2-mL samples were taken from the cultures and immediately centrifuged (2 min, 13,000 rpm, 4 °C). The resulting cell pellets were washed twice with fresh isoosmotic minimal medium to remove external αGG. The obtained pellets were suspended in 1 mL of methanol and incubated for 20 min at 70 °C to extract low molecular mass compounds. The dry residue was treated with 0.5 mL chloroform and 1 mL water to remove membranes, pigments and soluble proteins. After centrifugation, the aqueous phase was taken and dried in a vacuum concentrator. In order to remove high concentrations of salts and add the internal standard ribitol, the dry residue was dissolved in 80 µL absolute ethanol containing 200 µg mL^−1^ ribitol. After centrifugation, the liquid phase was transferred into a new vial, dried and stored at room temperature until analysis of the αGG concentrations by GC was performed as described [46].

### DNA isolation, transfer, and manipulations

Standard procedures were employed for plasmid isolation, cloning, and transformation of *E. coli* DH5α, as well as for electrophoresis [44]. Transformation of *C. glutamicum* was performed by electroporation using the methods described in Ref. [47], the recombinant strains were selected on LB-BHIS agar plates containing kanamycin (25 µg mL^−1^) and/or spectinomycin (30 µg mL^−1^). Electroporation of *E. coli* was performed using the methods mentioned in the Ref. [48]. All enzymes used were obtained from New England Biolabs and used according to the instructions of the manufacturer. PCR experiments were performed in a Tprofessional thermocycler (Biometra). Deoxynucleotide triphosphates were obtained from Bio-Budget, oligonucleotides (primers) from Eurofins MWG Operon (Table 2). Cycling times and temperatures were chosen according to fragment length and primer constitution. PCR products were separated on agarose gels and purified using the Nucleospin extract II kit (Macherey & Nagel). All cloned fragments were verified by sequencing (GATC Biotech AG).

**Table 2:** Oligonucleotides used in this study

Oligonucleotide	Sequence	Purpose
ggpS_5′_cgl	5′-GCCTGCAGAGGAGAGGGCAAAAAATGAATTCATCCC-3′	pEKEx2-GgpPS
ggpS_3′_cgl	5′-GCGGATCCTCACTTCACAGGTCAAGCTTAT-3′	pEKEx2-GgpPS
ggpP_5′_cgl	5′-GCGGTACCAGGAGGAGGCTAATAATGGTATTACACCAACAAC-3′	pEKEx2-GgpP
ggpP_3′_cgl	5′-GCGAATTCTTGCTAGTGGTGGTGGTGGTGGTGCTGGGAAAAA TGGACTCTTCGGCGCTGGGCCGCCTGCT-3′	pEKEx2-GgpP
otsA-for	5′-GCTCTAGAGCATGGATGATTCCAATAGCTT-3′	pASK-IBA3-*otsA*
otsA-rev	5′-GCCCTCGAGCGGTGAGTTTTCTCCCGACTGTG-3′	pASK-IBA3-*otsA*
glgA-IM-for	5′-TGATGCGGCCGACAAATG-3′	pDrive-IMglgA
glgA-IM-rev	5′-TGCGTGAGATCGCTGAAG-3′	pDrive-IMglgA, analysis of *glgA* inactivation
M13-FP	5′-TGTAAAACGACGGCCAGT-3′	analysis of *glgA* inactivation

### Heterologous expression of *ggpS* and *ggpP* in *C. glutamicum*

For IPTG-inducible heterologous expression of *ggpS* and *ggpP* the vectors pEKEx2 and pEKEx3 were used. The *ggpP* gene was amplified via PCR from genomic DNA of *Synechocystis* sp. PCC6803 using the oligonucleotide primers ggpP_5′_cgl and ggpP_3′_cgl. Using the PCR-generated *Kpn*I and *EcoR*I restriction sites of PCR product, the 1564 bp PCR product was ligated into pEKEx2 and transformed into *E. coli*. The resulting plasmid pEKEx2-*ggpP* was isolated from *E. coli.* The *ggpS* gene was amplified including the strep-tag coding sequence from the plasmid pASK-IBA-3-ggpS-strep using the oligonucleotide primers ggpS_5′_cgl and ggpS_3′_cgl. Using the PCR-generated *Pst*I and *BamH*I restriction sites of PCR product, the 1570 bp PCR product was ligated into pEKEx2-ggpS and transformed into *E. coli*. The resulting plasmid pEKEx2-*ggpSP* was isolated from *E. coli* and the nucleotide sequence controlled by sequencing (GATC Biotech). For heterologous expression of *ggpS* and *ggpP* in kanamycin-resistant *C. glutamicum* integration mutants the plasmid pEKEx3-*ggpSP* was constructed. For this purpose, the 5013 bp fragment carrying *ggpP* and *ggpS* was excised from pEKEx2-*ggpSP* using XhoI and PstI and cloned into pEKEx3 cut with the same enzymes, and transformed into *E. coli*. The resulting plasmid pEKEx3-*ggpSP* was isolated from *E. coli* and the nucleotide sequence controlled by sequencing.

### Inactivation of *glgA* in *C. glutamicum* strains

Inactivation of the chromosomal *glgA* gene (orf *cg1268*) in *C. glutamicum* WT and *C. glutamicum* Δ*otsA* was performed essentially as described for the inactivation of the *pgm* gene [38], using the plasmid pDrive-IMglgA. This plasmid was constructed by PCR-amplification of a DNA fragment covering nucleotides 85–636 of the annotated *glgA* gene, using primers glgA-IM-for and glgA-IM-rev (Table 1). The 551 bp PCR product was directly cloned into the TA-cloning vector pDrive (Qiagen) according to the manufacturer’s instructions and the resulting vector pDrive-IMglgA transformed into *E. coli* DH5α. After isolation of the recombinant plasmid, it was electroporated into *C. glutamicum* WT and *C. glutamicum* Δ*otsA*. Integration of pDrive-IMglgA at the genomic *glgA* locus in *C. glutamicum* and thus inactivation of the *glgA* gene was confirmed by PCR using primers glgA-IM-rev and M13-FP resulting in a specific 683 bp product for *C. glutamicum* IM*glgA* and *C. glutamicum* Δ*otsA* IM*glgA*.

### Overproduction, purification, and activity measurements for the trehalose 6-phosphate synthase OtsA from *C. glutamicum*

For heterologous expression in *E. coli*, the *otsA* gene of *C. glutamicum* was amplified from genomic DNA by PCR using the primers otsA-fw and otsA-rv (Table 1) and the resulting fragment cloned into the vector pASK-IBA3 (IBA GmbH, Göttingen Germany), according to the supplier’s manual. Expression of *otsA*-*strep* was induced by the addition of anhydrotetracycline (2 µg mL^−1^ at an OD600 of 1, and cells were harvested 6 h later by centrifugation at 5000×*g* for 5 min. The cells were suspended in wash buffer (100 mM Tris–HCl, pH. 8.0, and 150 mM NaCl) and disrupted by passing three times through a French pressure cell press (SLM-AMINCO) at 18,000 psi. After centrifugation at 15,000×*g* for 20 min, the supernatant was subjected to Strep-tag purification (IBA GmbH) according to the supplier’s manual. The purified OtsA enzyme was frozen in liquid nitrogen and stored at − 80 °C until further use.

The activity of purified OtsA was determined using an assay similar to the assay recently described for the determination of GgpS activity [20]. In detail, 0.2 µg of purified OtsA were incubated in a total volume of 80 µL at 30 °C for 10 min in assay buffer (10 mM TRIS-maleate, 5 mM MgCl_2_, 0.05 mM EDTA, pH 7.5) in presence of 3 mM of UDP-glucose or ADP-glucose and 3 mM of glucose-6-phosphate as substrates. The reaction was stopped by incubation for 10 min at 95 °C and formed trehalose-6-phosphate was then dephosphorylated by addition of 1 U alkaline phosphatase (FastAP, Fermentas, St. Leon-Roth, Germany) and incubation for 30 min at 30 °C. Sample preparation for gas chromatography and analyses of trehalose concentrations by split injection capillary gas chromatography using xylitol as internal and trehalose as external standard were performed as described by Novak et al. [20] and Wolf et al. [37], respectively.

### Protein analysis

Protein concentrations were determined using the Roti-Nanoquant kit (Roth) with bovine serum albumin as the standard. SDS-PAGE was performed according to Laemmli [49]. Standard loading buffer (4×) contained 8% (w/v) SDS, 20% (v/v) glycerol, 10 mM EDTA, 100 mM Tris/HCl, pH 6.8, 2% (v/v) β-mercaptoethanol, and 1 mg bromphenol blue mL^−1^. Western blot experiments for the detection of His-tagged GgpP was performed as described for GlgX–His [39]. Detection of the Streptavidin-tagged GgpS protein and determination of GgpS activity in cell free extracts of *C. glutamicum* strains were performed as recently described [20].

## Results

### NaCl-triggerd αGG synthesis in *C. glutamicum* (pEKEx2-*ggpSP*)

**Fig. 1:**
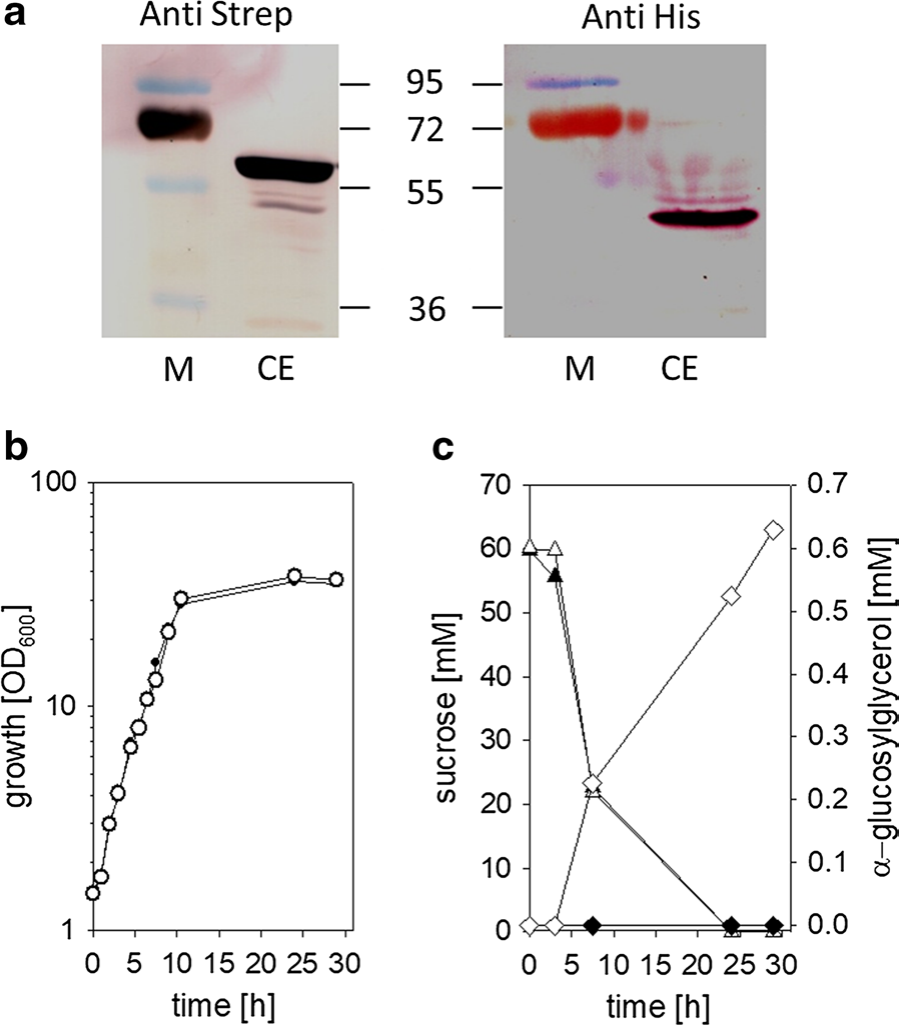
Heterologous expression of *ggpS* and *ggpP* from *Synechocystis* sp. PCC 6803 enable NaCl-triggerd αGG synthesis in *C. glutamicum* (pEKEx2-*ggpSP*). **a** Analysis of cell free extracts (CE) of *C. glutamicum* (pEKEx2-*ggpSP*) for expression of strep-tagged GgpS and his-tagged GgpP by Western immunoblot with antibodies against the tags; protein sizes (kDa) are indicated for markers (M) bands **a**. Growth (circles) **b**, substrate consumption (triangles) and αGG fromation (diamonds) **c** of *C. glutamicum* (pEKEx2-*ggpSP*) during cultivation in CgC-minimalmedium with initially 1% sucrose in absence (solid symbols) and presence of 750 mM NaCl (open symbols). NaCl was added to the culture after 4 h of cultivation. Data from one representative experiment of a series of three are shown

**Fig. 2:**
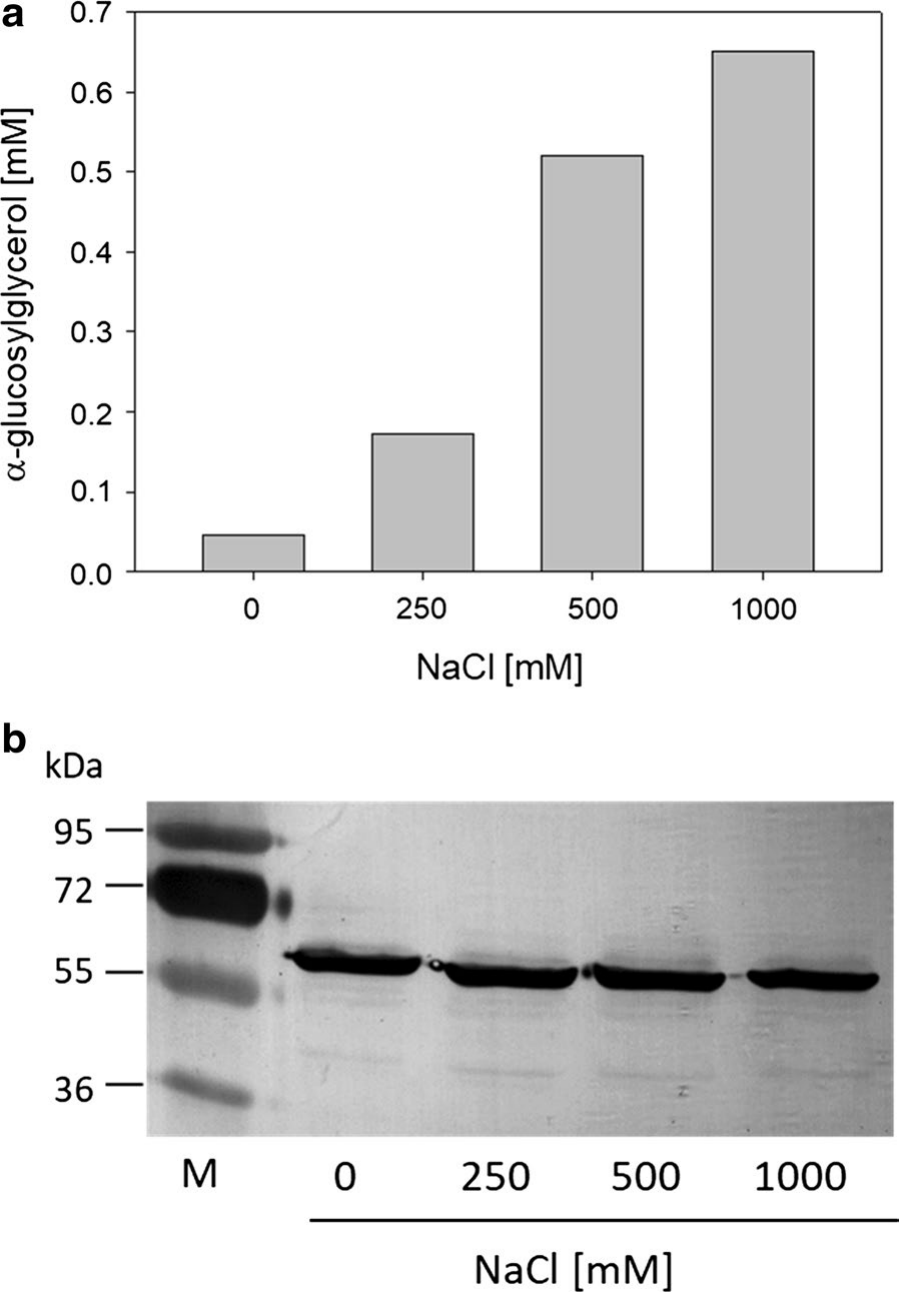
Analysis of the NaCl dependency of αGG synthesis in *C. glutamicum* (pEKEx2-*ggpSP*). αGG concentrations in culture supernatants after 24 h cultivation of *C. glutamicum* (pEKEx2-*ggpSP*) in minimal medium with initially 1% sucrose as sole carbon source **a**. NaCl was added in indicated concentrations after 4 h of cultivation. Analysis of cell free extracts (CE) of *C. glutamicum* (pEKEx2-*ggpSP*) cultivated at different NaCl concentrations for expression of strep-tagged GgpS by Western immunoblot with antibodies against the tag **b**; protein sizes (kDa) are indicated for markers (M) bands

The compatible solute αGG is synthesized in *Synechocystis* sp. PCC 6803 in a two-step reaction in which the enzymatic condensation of ADP-glucose and glycerol 3-phosphate by GG-phosphate synthase (GGPS) is followed by the dephosphorylation of the intermediate by the GG-phosphate phosphatase (GGPP). The genes *ggpS* and *ggpP* from *Synechocystis* sp. PCC 6803 were cloned into the expression plasmid pEKEx2 and the resulting plasmid was introduced into *C. glutamicum*. Presence of the plasmid encoded enzymes in extracts of *C. glutamicum* (pEKEx2-*ggpSP*) was shown by western blot analysis using antibodies raised against the C-terminal His-Tag of GgpP and the C-terminal Strep-Tag of GgpS (Fig. 1a), and a GgpS activty of 9.52 ± 2.06 µmol min^−1^ (mg protein)^−1^ was observed in cell free extracts of *C. glutamicum* (pEKEx2-*ggpSP*). However, no formation of αGG was observed in samples from the culture supernatant of *C. glutamicum* (pEKEx2-*ggpSP*) during cultivation in minimal medium with 1% (w/v) sucrose (Fig. 1b, c). GgpS from *Synechocystis* sp. PCC 6803 underlies a post-translational regulatory mechanism, which is based on salt-dependent electrostatic interaction of the protein with the negatively charged backbone of polyanions, e.g. DNA and RNA) [20]. This mode of activity control is probably conserved even when the gene is heterologously expressed in *C. glutamicum*. Indeed the GgpS activity in extracts of *C. glutamicum* (pEKEx2-*ggpSP*) increased to 27.64 ± 1.3 µmol min^−1^ (mg protein)^−1^ when 200 mM NaCl was present in the assay buffer. To activate the GgpS in *C. glutamicum* (pEKEx3-*ggpSP*) the cells were stressed during cultivation by addition of NaCl (final concentration 750 mM). Accumulation of αGG was obserevd exclusively in the culture supernatants sampled after the addition of NaCl (Fig. 1b, c), after 25 h cultivation 0.62 ± 0.07 mM αGG was formed. More detailed experiments with *C. glutamicum* (pEKEx2-*ggpSP*) showed the dependence of externally accumulated αGG on the NaCl concentration during cultivation, an increase of the external NaCl concentrations caused increased formation of αGG (Fig. 2a). No variations of GgpS amounts were detected in western blot analyses with an antibody against the C-terminal Strep-Tag of GgpS, when cells extracts of *C. glutamicum* (pEKEx2-*ggpSP*) from the cultivations at different amounts of NaCl were analyzed (Fig. 2b).

NaCl-dependent accumulation of αGG in supernatants of *C. glutamicum* (pEKEx2-*ggpSP*) depends on both intracellular synthesis of αGG and αGG export. Intracellular αGG was analysed in cells of *C. glutamicum* (pEKEx2-*ggpSP*) in samples taken from cultivations 6 h after the addition of 600 mM NaCl and from untreated cultures. Intracellular αGG concentration of about 12 mM and 450 mM were measured for cells of *C. glutamicum* (pEKEx2-*ggpSP*) from untreated and NaCl-shocked cultivations, respectively. This results indicates that in absence of NaCl-stress the GgpS activity was low, whereas the addition of NaCl to the medium indeed activated intracellular αGG synthesis by the GgpS.

Taken together these data show, that upon NaCl-induced stress GgpS is activated in *C. glutamicum* (pEKEx2-*ggpSP*) similar as in *Synechocystis* sp. PCC 6803 leading to the synthesis of αGG, which is then exported to the culture medium different from the situation in *Synechocystis* sp. PCC 6803.

### Optimization of precursor supply for efficient αGG synthesis in *C. glutamicum*

GgpS of *Synechocystis* sp. PCC 6803 requires ADP-glucose as a substrate and does not utilize alternative activated sugars such as UDP-glucose [50]. The precursor for αGG synthesis ADP-glucose is a key intermediate of glycogen synthesis. In *C. glutamicum* ADP-glucose is formed by the *glgC* encoded ADP-glucosepyrophosphoralyse and used for the elongation of glucan chains by the *glgA* encoded glycogen synthase [40, 41]. The genes *glgC* and *glgA*, are situated adjacent on the genome sequence of C*. glutamicum*, separated by 153 bp, and are divergently transcribed [51]. Thus inactivation of *glgA* by insertion mutagenesis is possible in *C. glutamicum* without affecting *glgC* [36]. The gene *glgA* was inactivated by insertion of the vector pDrive, the resulting strain *C. glutamicum* IM*glgA*did not accumulate glycogen during cultivation in minimal medium with either 1% (w/v) glucose or 1% (w/v) sucrose as substrate (data not shown). For heterologous expression of *ggpS* and *ggpP* the plasmid pEKEx3-*ggpSP* was introduced into *C. glutamicum* IM*glgA*. During cultivation in minimal medium with 2% (w/v) sucrose and 750 mM NaCl the resulting strain *C. glutamicum* IM*glgA* (pEKEx3-*ggpSP*) accumulated within 24 h of cultivation 3.23 ± 0.15 mM αGG in its supernatant, which is about 6 times more than the amount of 0.57 ± 0.06 mM αGG observed for the parental strain *C. glutamicum* (pEKEx3-*ggpSP*) (Table 3). The initially provided carbon source sucrose was completely consumed by both strains, *C. glutamicum* IM*glgA* (pEKEx3-*ggpSP*) formed about 2.3 g L^−1^ less biomass than *C. glutamicum* (pEKEx3-*ggpSP*).

**Table 3:** Production of α-GG with different *C. glutamicum* strains carrying the plasmid pEKEx3-*ggpPS* during cultivation in CgC minimal medium with 1% (w/v) sucrose as sole substrate

Strain	αGG production (mM)	Biomass (g cdm L^−1^)	Substrate consumption (mM)	Yield P/S (mmol mol l^−1^)
*C. glutamicum* (pEKEx3-*ggpSP*)	0.57 ± 0.06	7.60 ± 0.03	60.82 ± 1.59	9.4 ± 1.1
*C. glutamicum* IM*glgA* (pEKEx3-*ggpSP*)	3.23 ± 0.15	5.31 ± 0.31	62.23 ± 2.63	52.0 ± 2.4
*C. glutamicum* Δ*otsA* (pEKEx3-*ggpSP*)	2.19 ± 0.20	4.91 ± 0.19	64.78 ± 2.74	33.8 ± 3.1
*C. glutamicum* IM*glgA* Δ*otsA* (pEKEx3-*ggpSP*)	4.22 ± 0.22	4.31 ± 0.23	61.19 ± 3.98	69.0 ± 3.6

Strains were inoculated at an OD_600_ of 1, α-GG synthesis was induced by addition of NaCl (final concentration 750 mM) after 4 h of cultivation. The fermentations were performed in triplicate, samples of each fermentation were analyzed in triplicate, means are from the three independent experiments; the indicated experimental error means standard deviation

Besides for glycogen synthesis ADP-glucose is also used in some bacteria such as *Propionibacterium freudenreichii* for trehalose synthesis. Usually the trehalose-6-phosphate synthase OtsA uses UDP-glucose as substrate [52], but the enzyme from *P. freudenreichii* prefers ADP-glucose over UDP-glucose [53]. In *C. glutamicum* OtsA contributes to trehalose synthesis [36, 37], the substrate spectrum of OtsA from *C. glutamicum* has not been analyzed. For biochemical characterization of *C. glutamicum* OtsA the gene *otsA* was cloned into the vector pASK-IBA3+. Thereby a gene fusion was generated, which encodes a C-terminal Strep-tagged OtsA variant for purification via affinity chromatography. For in vitro characterization of the substrate specificity of the 53 kDa OtsA-Strep construct, the protein was purified via Strep-Tactin affinity chromatography (Additional file 1: Fig. S1). Specific activities of 0.29 ± 0.01 µmol min^−1^ (mg protein)^−1^ and 0.15 ± 0.01 were determined when 5 mM of UDP-glucose and ADP-glucose, respectively, were used as substrate for purified OtsA-Strep. Deletion of *otsA* improved αGG production of the strain *C. glutamicum* ΔotsA (pEKEx3-*ggpSP*) when compared to *C. glutamicum* (pEKEx3-*ggpSP*) (Table 3). This indicates that supply of the precursor ADP-glucose was improved by deletion of *otsA* in *C. glutamicum.* Taken together it can be concluded that *C. glutamicum* OtsA used UDP- as well as ADP-glucose as precursor for trehalose-phosphate synthesis and therefore *otsA* is an important target for the metabolic engineering towards improved αGG synthesis.

In a next step *glgA* was inactivated by insertion mutagenesis in *C. glutamicum* ΔotsA. Upon plasmid encoded overexpression of *ggpS* and *ggpP* the resulting strain *C. glutamicum* ΔotsA IMglgA (pEKEx3-*ggpSP*) formed during cultivation in minimal medium with 2% (w/v) sucrose and 750 mM NaCl 4.22 ± 0.22 mM αGG, which is more than the amounts of αGG formed by each of the parental strains (Table 3). As this increased product formation by *C. glutamicum* ΔotsA IMglgA (pEKEx3-*ggpSP*) is achieved by the use of nearly the same amount of substrate, the yield (p/s) is further increased when compared to the predecessor strains (Table 3).

**Fig. 3:**
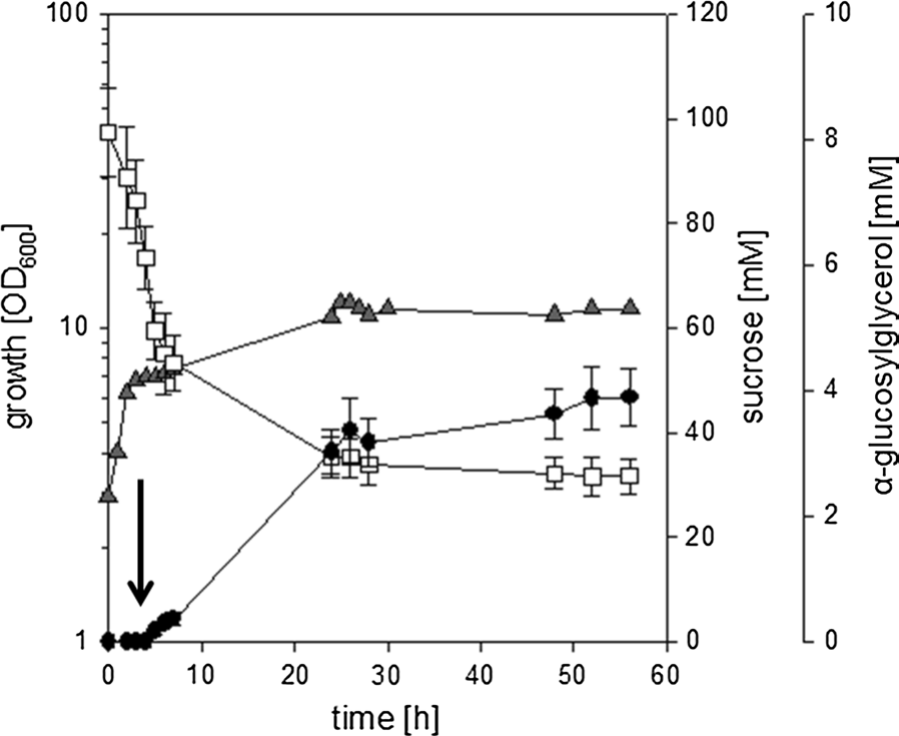
αGG accumulation during cultivation of *C. glutamicum* IM*glgA* Δ*otsA* (pEKEx3-*ggpSP*) in small-scale bioreactors in CgC minimal medium with 2% sucrose. The culture was inoculated at an OD_600_ of about 1, after 4 h of cultivation a hyperosmotic shock was applied by addition of NaCl (final concentration 750 mM; the timepoint of NaCl addition is indicated by a black arrow). Growth (grey triangles), sucrose concentration (white squares), and α-GG concentration (black circles) were analyzed throughout cultivation. The fermentation was performed in duplicates, for substrate and product concentrations samples from each fermentation were analyzed in triplicates. Means of substrate and product concentrations are from two independent experiments; error bars indicate standard deviations. For growth a representative curve of the two experiments is shown

The properties of the strain *C. glutamicum* ΔotsA IMglgA (pEKEx3-*ggpSP*) were also tested in batch-fermentations in small, pH-controlled, aerated 1 L bioreactors in CgC-minimal medium with 2% (w/v) sucrose as sole source of carbon and energy. As depicted in Fig. 3, growth of *C. glutamicum* ΔotsA IMglgA (pEKEx3-*ggpSP*) slowed down and production of αGG set in after 4 h of cultivation, when the NaCl (final concentration 750 mM) was added to the culture. Production of αGG, slow growth and concomitant consumption of sucrose continued until a concentration of about 3.4 ± 0.5 mM αGG was formed, which was only slightly increased to 3.9 ± 0.5 mM upon prolonged incubation. After about 20 h of cultivation both growth and αGG production ceased nearly completely albeit a concentration of about 35.4 ± 3.4 mM of the substrate sucrose was still present in the culture broth.

Taken together these results show, that upon heterologous expression of the genes *ggpS* and *ggpP* for αGG synthesis from *Synechocystis* sp. PCC 6803 *C. glutamicum* produces and secretes the compatible solute αGG in response to NaCl-induced hyperosmotic stress. Moreover, genetic modifications of *C. glutamicum* strains for an improved supply of the precursor ADP-glucose by inactivation of *glgA* and *otsA* led to an increased αGG production.

### Optimization of nitrogen-supply for enhanced αGG production by *C. glutamicum* ΔotsA IMglgA (pEKEx3-*ggpSP*)

**Fig. 4:**
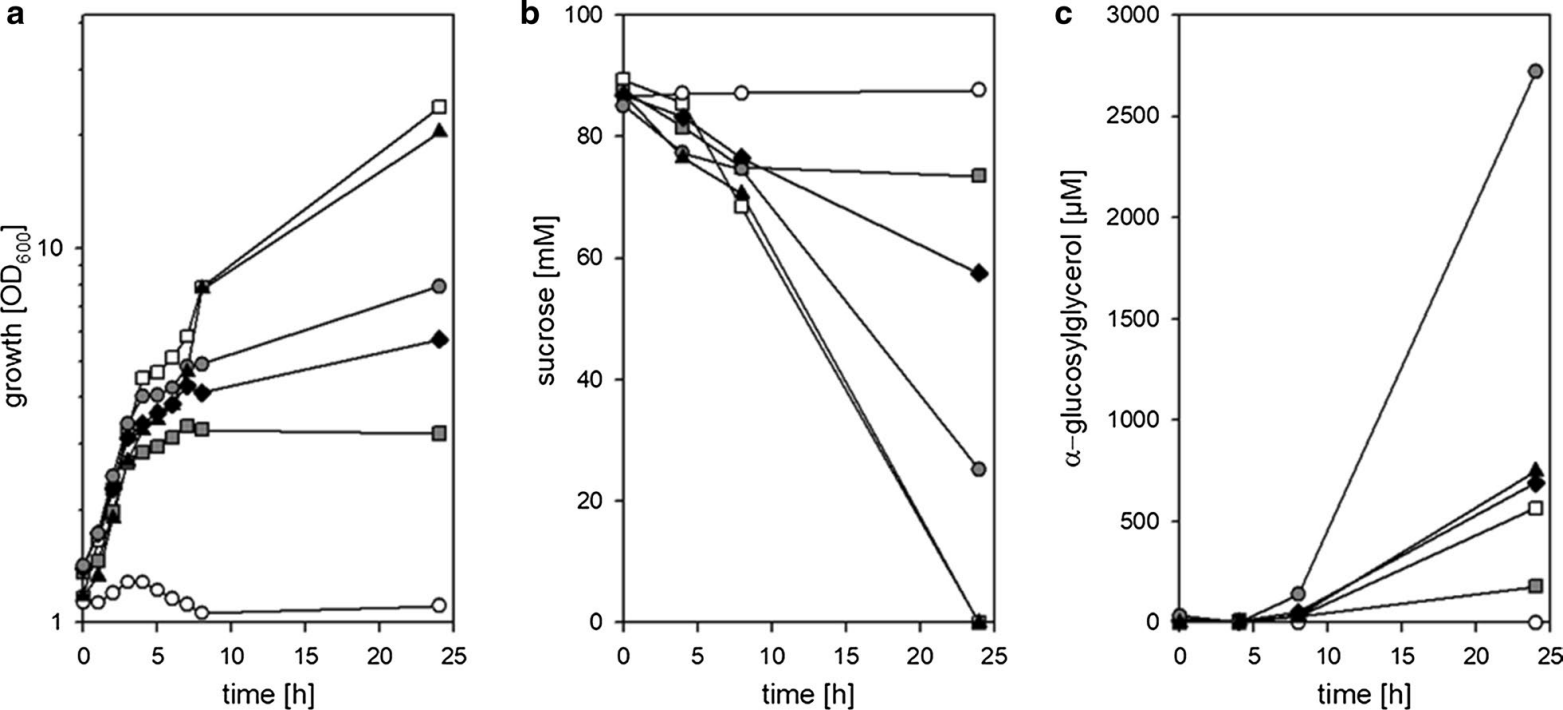
Effect of nitrogen limitation on α-GG production with *C. glutamicum* (pEKEx3-*ggpSP*). Growth **a**, substrate consumption **b** and αGG accumulation **c** were determined for *C. glutamicum* (pEKEx3-*ggpSP*) in the course of cultivation in CgC-minimal medium (open squares), or CgC-minimal medium without ammonia and 0 g L^−1^ urea (white circles), 0.1 g L^−1^ urea (grey squares), 0.5 g L^−1^ urea (solid diamonds), 1.0 g L^−1^ urea (grey circles), or 5.0 g L^−1^ urea (solid triangles). Data from one representative experiment of a series of three are shown

The production of αGG by *C. glutamicum* (pEKEX3-*ggpSP*) correlated well with the amount of externally present NaCl (Fig. 2). This observation fits well to the proposed mechanism for GgpS activation by increased intracellular of sodium ions [20]. Accumulation of compatible solutes is the common way in bacteria to respond to increased sodium concentrations, which in *C. glutamicum* proceeds in absence of external compatible solutes via synthesis of, e.g. proline and trehalose [37]. Synthesis of trehalose is abolished in OtsA and GlgA deficient *C. glutamicum* strains such as *C. glutamicum* ΔotsA IMglgA [36], however depending of the availability of suitable nitrogen-sources proline is accumulated as main compatible solute [37]. As intracellular proline accumulation might counteract the conditions required for efficient αGG synthesis, effects of limited nitrogen availability on αGG production with *C. glutamicum* strains were tested. To optimize nitrogen supply for an optimal GG production in the presence of still sufficient growth, *C. glutamicum* (pEKEx3-*ggpSP*) was cultivated in CgC-minimal medium without ammonium sulfate and varying amounts of urea (0; 0.1, 0.5, 1.0, 5 g L^−1^). NaCl (final concentration 750 mM) was added to the cultures after 4 h of cultivation to induce αGG production. Neither growth nor αGG production were observed for cultivations in the medium without urea (Fig. 4a). In medium containing 5 g L^−1^ urea growth (Fig. 4a), sucrose utilization (Fig. 4b), and αGG production (Fig. 4c) proceeded as observed for CgC-minimal medium. Also for the media containing 0.1, 0.5, and 1.0 g L^−1^ urea growth of *C. glutamicum* (pEKEx3-*ggpSP*) was observed. The final ODs increased in parallel to the increase of urea in the medium, but the final ODs of these cultures remained significantly below the final ODs observed for the cultivations with 5.0 g L^−1^ urea and in CgC minimal medium, which contains 5 g L^−1^ urea plus 5 g L^−1^ ammonia chloride as nitrogen sources (Fig. 4a). This result indicates that different levels of N-limitation were installed in these cultivations. Concerning the production of αGG in N-limited cultures, the highest amount of 2.7 mM was produced in cultivations of *C. glutamicum* (pEKEx3-*ggpSP*) with 1.0 g L^−1^ urea (Fig. 4c); however, the initially provided sucrose was not completely consumed after 24 h of cultivation (Fig. 4b).

Effects of nitrogen limitation on the αGG production were also tested for the strains with optimized provision of the precursor ADP-glucose. As depicted in Table 4 αGG production after the hyperosmotic shock by NaCl addition was significantly increased for all strains tested, e.g. *C. glutamicum* ΔotsA IMglgA (pEKEx3-*ggpSP*) produced 6.40 ± 0.88 mM αGG in the cultivations with 1.0 g L^−1^ urea, which is nearly twice the amount of the αGG produced in cultivations in CgC minimal medium (Table 3). As all tested strains consumed in nitrogen limited cultivations only about half of the amount of sucrose they consumed in non-limited cultivations, the αGG yield (product/substrate) increased significantly (Tables 3, 4).

**Table 4:** Production of α-GG with different *C. glutamicum* strains carrying the plasmid pEKEx3-*ggpPS* during cultivation minimal medium with 1% (w/v) sucrose as sole substrate and 1.0 g L^−1^ urea as sole nitrogen source

Strain	αGG production (mM)	Biomass (g cdm L^−1^)	Substrate consumption (mM)	Yield P/S (mmol mol^−1^)
*C. glutamicum* (pEKEx3-*ggpSP*)	2.69 ± 0.46	2.34 ± 0.06	38.04 ± 0.98	70.9 ± 14.0
*C. glutamicum* IM*glgA* (pEKEx3-*ggpSP*)	5.24 ± 0.57	1.80 ± 0.01	35.70 ± 5.90	146.6 ± 16.1
*C. glutamicum* Δ*otsA* (pEKEx3-*ggpSP*)	5.54 ± 0.60	2.39 ± 0.03	35.24 ± 3.13	157.4 ± 17.0
*C. glutamicum* IM*glgA* Δ*otsA* (pEKEx3-*ggpSP*)	6.40 ± 0.88	2.20 ± 0.01	34.41 ± 4.36	186.1 ± 25.5

Strains were inoculated at an OD_600_ of 1, α-GG synthesis was induced by addition of NaCl (final concentration 750 mM) after 4 h of cultivation. The fermentations were performed in triplicate, samples of each fermentation were analyzed in triplicate, means are from the three independent experiments; the indicated experimental error means standard deviation

**Fig. 5:**
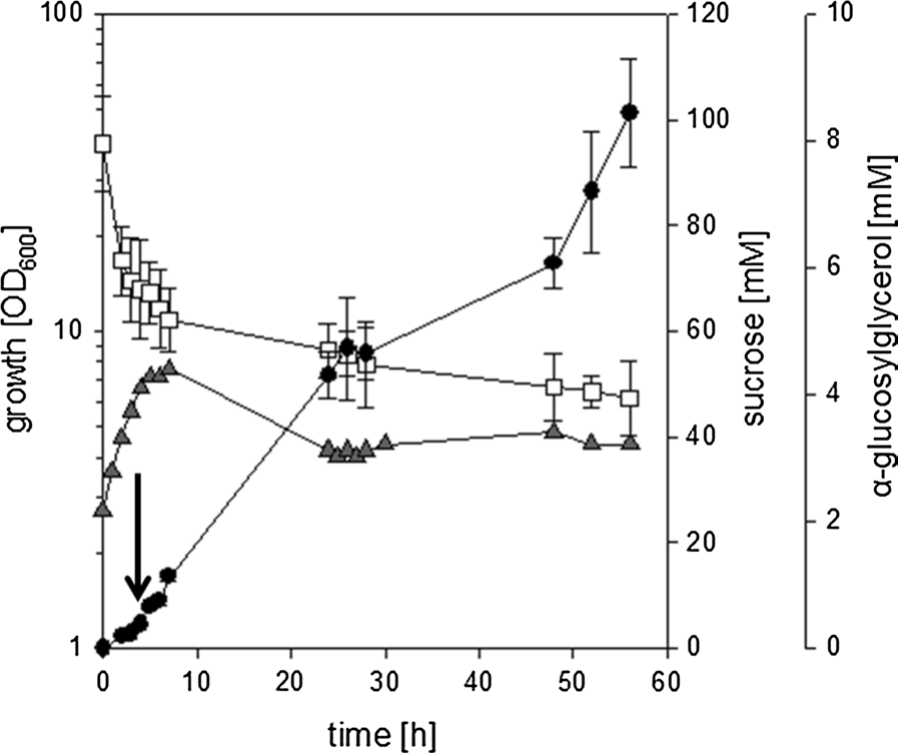
Effect of nitrogen limitation on αGG accumulation during cultivation of *C. glutamicum* IM*glgA* Δ*otsA* (pEKEx3-*ggpSP*) in small-scale bioreactors in CgC minimal medium with 2% sucrose. The culture was inoculated at an OD_600_ of about 1, after 4 h of cultivation a hyperosmotic shock was applied by addition of NaCl (final concentration 750 mM; the timepoint of NaCl addition is indicated by a black arrow). Growth (grey triangles), sucrose concentration (open squares), and α-GG concentration (solid circles) were analyzed throughout cultivation. The fermentation was performed in duplicates, for substrate and product concentrations samples from each fermentation were analyzed in triplicates. Means of substrate and product concentrations are from two independent experiments; error bars indicate standard deviations. For growth a representative curve of the two experiments is shown

The effects of N-limitation on αGG production with the strain *C. glutamicum* ΔotsA IMglgA (pEKEx3-*ggpSP*) were also tested in batch-fermentations in a small, pH-controlled, aerated bioreactor in a 1-L scale in minimal medium with 1.0 g L^−1^ urea as sole nitrogen source and 2% (w/v) sucrose as sole source of carbon and energy. As described above for conditions of sufficient nitrogen supply, growth of *C. glutamicum* ΔotsA IMglgA (pEKEx3-*ggpSP*) slowed down and production of αGG set in after 4 h of cultivation, when NaCl (final concentration 750 mM) was added to the culture (Fig. 5). In difference to the cultivation in CgC minimal medium after prolonged incubation the biomass in the culture liquid significantly decreased. This decrease coincided with the formation of foam and especially cell aggregates in the bioreactor, which occurred even though anti-foam reagent was present in the cultivation broth. Biomass was found sticking to all components (e.g. stirrer, gas inlet, and electrodes) and the glass wall in the headspace of the fermenter. Despite the loss of biomass in the culture liquid production of αGG and concomitant sucrose consumption slowly continued until the cultivation was ended 58 h after the inoculation, when a final concentration of 8.4 ± 0.9 mM αGG was present in the culture liquid. In the course of the fermentation 48.2 ± 8.1 mM sucrose have been consumed in total, which results in a yield of about 0.17 mol mol^−1^. These results show that by combining strain development with optimized cultivation conditions such as nitrogen limitation efficient αGG production with recombinant *C. glutamicum* strains was possible. However, the high amounts of residual sucrose observed here during cultivations of *C. glutamicum* Δ*otsA* IM*glgA* (pEKEx3-*ggpSP*) exclusively in stirred bioreactors (Figs. 3, 5) and especially the strong formation of foam and cell aggregates observed for N-limited cultures indicate, that mechanical robustness of the cells might be compromised by the here applied strategies to limit synthesis of compatible solutes.

### Analysis of αGG production in the trehalose synthesis deficent strain *C. glutamicum* Δ*otsA*Δ*treY*Δ*treS* (pEKEx3-*ggpSP*)

Beside its role as a compatible solute trehalose is in *C. glutamicum* a precursor for the synthesis of trehalose mycolates [36, 37], which are important building blocks of the mycolic acid layer, a second permeability barrier functionally similar to the Gram-negative outer membrane [54, 55]. For the trehalose synthesis and thus mycolic acid layer deficient strain *C. glutamicum* Δ*otsA*Δ*treY*Δ*treS* besides increased osmo-sensitivity and aggregation of cells in liquid culture was reported [56]. Inactivation of *glgA* impairs synthesis of glycogen, which acts as precursor for trehalose synthesis via the TreYZ pathway [36]. Thus redirection of the carbon flux in *C. glutamicum* Δ*otsA* IM*glgA* (pEKEx3-*ggpSP*) probably resulted in a lack of trehalose, which is required for the synthesis of the mycolic acid layer. The increased sensitivity to osmotic stress and tendency to form cell aggregates might underlie the in-complete substrate utilization and loss of biomass observed during cultivation in stirred bioreactors. To rule out that trehalose-synthesis deficiency is the reason for the improved αGG production of *C. glutamicum* Δ*otsA* IM*glgA* (pEKEx3-*ggpSP*) when compared to *C. glutamicum* IM*glgA* (pEKEx3-*ggpSP*) or *C. glutamicum* Δ*otsA* (pEKEx3-*ggpSP*) (Tables 3, 4), αGG production of *C. glutamicum* Δ*otsA*Δ*treY*Δ*treS* (pEKEx3-*ggpSP*) was analyzed. Accumulation of αGG in the culture broth and αGG productivity of *C. glutamicum* Δ*otsA*Δ*treY*Δ*treS* (pEKEx3-*ggpSP*) in cultivations in CgC-minimal medium as well as N-limited medium were significantly lower (Table 5) than the parameters determined for *C. glutamicum* Δ*otsA* IM*glgA* (pEKEx3-*ggpSP*) and quite similar to the parameters determined for *C. glutamicum* Δ*otsA* (pEKEx3-*ggpSP*) (Tables 3, 4), which is still able to synthesize trehalose [37]. Taken together these results show that indeed the strategy to increase availability of the precursor ADP-glucose by inactivation of *glgA* and deletion of *otsA* was responsible for the improved αGG by *C. glutamicum* Δ*otsA* IM*glgA* (pEKEx3-*ggpSP*) and not the concomitantly obtained trehalose-synthesis deficiency.

**Table 5:** Production of α-GG with the trehalose synthesis deficient strain *C. glutamicum* Δ*otsA* Δ*treY* Δ*treS* (pEKEx3-*ggpPS*) during cultivation with 1% (w/v) sucrose as sole substrate in CgC minimal medium or minimal medium and 1.0 g L^−1^ urea as sole nitrogen source

Medium	αGG production (mM)	Biomass (g cdm L^−1^)	Substrate consumption (mM)	Yield P/S (mmol mol l^−1^)
CgC-minimal medium	2.83 ± 0.37	4.33 ± 0.41	63.67 ± 2.86	44.40 ± 5.76
Minimal medium with 1.0 g L^−1^ urea	3.91 ± 0.48	1.85 ± 0.06	31.48 ± 1.36	124.23 ± 15.40

Cultures were inoculated at an OD_600_ of 1, α-GG synthesis was induced by addition of NaCl (final concentration 750 mM) after 4 h of cultivation. The fermentations were performed in triplicate, samples of each fermentation were analyzed in triplicate, means are from the three independent experiments; the indicated experimental error means standard deviation

## Discussion

The here developed microbial production of the compatible solute αGG using a genetically engineered *C. glutamicum* strain offers an alternative to the hitherto described enzymatic syntheses of this compound [15] and αGG production with an genetically engineered strain of *Synechocystis* sp. PCC 6803 [26]. Microbial synthesis of αGG allows a large flexibility for the use of different sources of carbon, which is not the case for the currently described processes for enzymatic αGG synthesis [22, 23]. The engineered *Synechocystis* sp. PCC 6803 strain produced about 1 mM of extracellular αGG during a production cycle of 6 days in a photobioreactor [26]. In this study more than 6 times higher αGG concentrations were obtained in cultivations of *C. glutamicum* Δ*otsA* IM*glgA* (pEKEx3-*ggpSP*) during 24 h, however sucrose was here used as source of carbon and energy. A drawback of the two microbial approaches to produce αGG is the need to induce αGG synthesis by addition of NaCl to the cultivation broth. Thus for further utilization of the produced αGG NaCl has to be removed in the course of the downstream processing. However, this technical problem of the current concept of αGG production with *C. glutamicum* strains overexpressing *ggpS* and *ggpP* from *Synechocystis* sp. PCC 6803 illustrates the intrinsic mode of activity control of the GGPS. Similar to the homologous situation in *Synechocystis* sp. PCC 6803 [20, 26] NaCl stress was shown to be required to initiate αGG synthesis in *C. glutamicum* (pEKEx3-*ggpSP*) cells, which contained the preformed αGG biosynthesis enzymes GGPS and GGPP. This result indicates that the mechanism of osmosensing of GGPS by salt-dependent protein-nucleic acid interactions recently described for the first time using in vitro studies [20] is conserved even when the enzyme is transferred into a heterologous host. Elimination of the intrinsic activity control of GgpS activity would probably cancel out the need of NaCl addition to the culture broth, which is currently required to start αGG synthesis. This approach should enable αGG production under normal osmotic conditions, however, the molecular basis underlying inhibition of GgpS activity by binding to DNA has not been elucidated so far.

In this context, the observed export of αGG is interesting in terms of mechanistic aspects. Under the applied experimental conditions, *C. glutamicum* secretes this compound into the surrounding medium, which raises at least two different questions, in view of the fact that αGG, although being a well-known compatible solute, is a foreign compound in the heterologous host, *C. glutamicum*: (i) By which kind of mechanism is αGG exported out of the *C. glutamicum* cell, and (ii) why is αGG continuously secreted albeit *C. glutamicum* could use it as compatible solute? In case of hypo-osmotic conditions, compatible solutes are in general expelled out of the cell via so-called mechanosensitive channels, and *C. glutamicum* possesses at least two of them, an MscS-type and an MscL-type channel [57, 58]. On first glance, it seems very unlikely that αGG uses this kind of pathway to leave the cell, since export of this compound was observed under hyperosmotic stress and mechanosensitive channels are closed under these conditions [59]. Consequently, it seems likely that αGG is secreted via a so far unknown solute export mechanism, most probably a general transport system devoted to the export of unwanted compounds out of the cytoplasm. The intracellular accumulation of 450 mM αGG indicates that export does not proceed very efficiently, which is not surprising as αGG is for *C. glutamicum* a foreign molecule neither synthesized nor consumed by this bacterium. On a second glance the high intracellular αGG concentration might allow also export of αGG via the mechanosensitive channels despite the hyperosmotic growth conditions. In *C. glutamicum* the mechanosensitive channel MscCG contributes under hyperosmotic stress conditions to the fine-tuning of the concentration of compatible solutes in the cytoplasm, which are accumulated, e.g. via the betaine transporter BetP [58]. According to the “pump and leak” model for the control of the steady state level of compatible solutes in the cytoplasm export of compatible solutes via MscCG compensates for their unbalanced uptake [58, 60]. In a similar manner the MscCG might export αGG when its intracellular accumulation requires to be compensated despite cultivation in presence of high NaCl concentrations.

The second question raised above is even more surprising. Considering the fact that *C. glutamicum* (pEKEx2-*ggpSP*) has to counteract hyperosmotic stress in the presence of an external concentration of 750 mM NaCl and is capable by synthesis of its genuine compatible solutes, e.g. proline or trehalose, to counteract this stress it seems surprising that αGG synthesis does not cease in the course of cultivation. In *Synechocystis* sp. PCC 6803 the physical stimulus for the synthesis of the compatible solute αGG is not osmotic stress: Synthesis of αGG exclusively proceeds in *Synechocystis* sp. PCC 6803 in response to ionic stress caused by addition of salts such as NaCl and not in response to addition of compounds like sucrose, which only increases the osmolality of the medium [46]. Different from the situation in *Synechocystis* sp. PCC 6803 addition of ionic compounds like NaCl as well as non-ionic compounds like sorbitol to the culture broth activate in *C. glutamicum* the hyperosmotic stress response, which in this bacterium comprises synthesis and/or uptake of compatible solutes [35] but also the uptake of potassium ions as an immediate reaction [61]. Taken together, the differences in the osmo- and ionic stress responses in *Synechocystis* sp. PCC 6803 and C. *glutamicum* might account for the continuous synthesis of αGG in *C. glutamicum* (pEKEx2-*ggpSP*) upon the addition of NaCl to the medium.

The export of αGG observed for the *C. glutamicum* strains overexpressing *ggpSP* allows an indirect insight into the capacity of *C. glutamicum* for glycogen synthesis. *C. glutamicum* transiently accumulates large amounts of glycogen when cultivated in presence of sugars [41], however the internal glycogen amount always depends on the rates for synthesis and degradation [38, 39]. Therefore αGG synthesis by GGPS and GGPP depends on the availability of the precursors glycerol 3-phosphate and ADP-glucose, which is the central intermediate of glycogen synthesis [62]. Availability of ADP-glucose dictates the rate of glycogen synthesis in bacteria as the rate of its synthesis by the ADP-glucose pyrophosphorylase is controlled in bacteria by multiple mechanisms [63, 64]. Redirection of the flux from glycogen synthesis to αGG synthesis by inactivation of *glgA* allows to assess a rate of ADP-glucose formation and thus precursor supply for glycogen synthesis in *C. glutamicum*, as αGG is constantly exported and not consumed. The continuous production of αGG reveals a continuous provision of the precursor ADP-glucose, which indicates the presence of a continuous flux towards glycogen synthesis in *C. glutamicum*. This consideration based on the continuously observed αGG accumulation fits within the proposed model of glycogen as a carbon capacitor in bacteria [38, 65–67]. The proportion of flux towards glycogen might be even slightly higher as here indicated by the synthesis of provision of αGG, as availability of the precursor glycerol 3-phosphate might here be limiting αGG synthesis. Taken together, the constant accumulation of αGG in the supernatant of *C. glutamicum* ΔotsA IMglgA (pEKEx3-*ggpSP*) indicates that a high proportion of the glycolytic flux can be channeled into glycogen, which vice versa is a prerequisite for a continuous supply of the precursor ADP-glucose allowing efficient microbial production of αGG in *C. glutamicum*.

## Additional file

**Additional file 1: Fig. S1.** Purification of recombinant streptavidin-tagged *C. glutamicum* OtsA from *E. coli* BL21(DE3)(pASK-IBA3-*otsA*): SDS-PAGE analysis of crude cell extract and Streptavidin-affinity chromatography flow-through, wash fraction, and elution fractions. PAGE ruler prestained protein ladder (MBI Fermentas) was used as marker.

## Abbreviations

cdm: cell dry mass
WT: wild-type
αGG: α-d-glucosylglycerol
