# Supplementary material for: Production of the compatible solute α-d-glucosylglycerol by metabolically engineered Corynebacterium glutamicum
Source: Microb Cell Fact. 2018 Jun 16;17:94. doi: 10.1186/s12934-018-0939-2 (PMC6004087; doi:10.1186/s12934-018-0939-2)
Supplement: Supplementary file 1 — Additional file 1: Fig. S1. Purification of recombinant streptavidin-tagged C. glutamicum OtsA from E. coli BL21(DE3)(pASK-IBA3-otsA): SDS-PAGE analysis of crude cell extract and Streptavidin-affinity chromatography flow-through, wash fraction, and elution fractions. PAGE ruler prestained protein ladder (MBI Fermentas) was used as marker. [file 12934_2018_939_MOESM1_ESM.docx]

Additional data to the manuscript:

### Production of the compatible solute α-D-glucosylglycerol by metabolically engineered *Corynebacterium glutamicum*

By Benjamin Roenneke, Natalie Rosenfeldt, Sami Derya, Jens F. Novak, Kay Marin, Reinhard Krämer, and Gerd M. Seibold

The supplementary data comprise one figure Fig. S1

Correspondence: Gerd M. Seibold, Institute of Microbiology & Biotechnology, University of Ulm, 89081 Ulm, Germany. Tel.: +49 (0)731 502 4853; fax: +49 (0)731 502 2719; e-mail: [gerd.seibold@uni-ulm.de](mailto:gerd.seibold@uni-ulm.de)

**
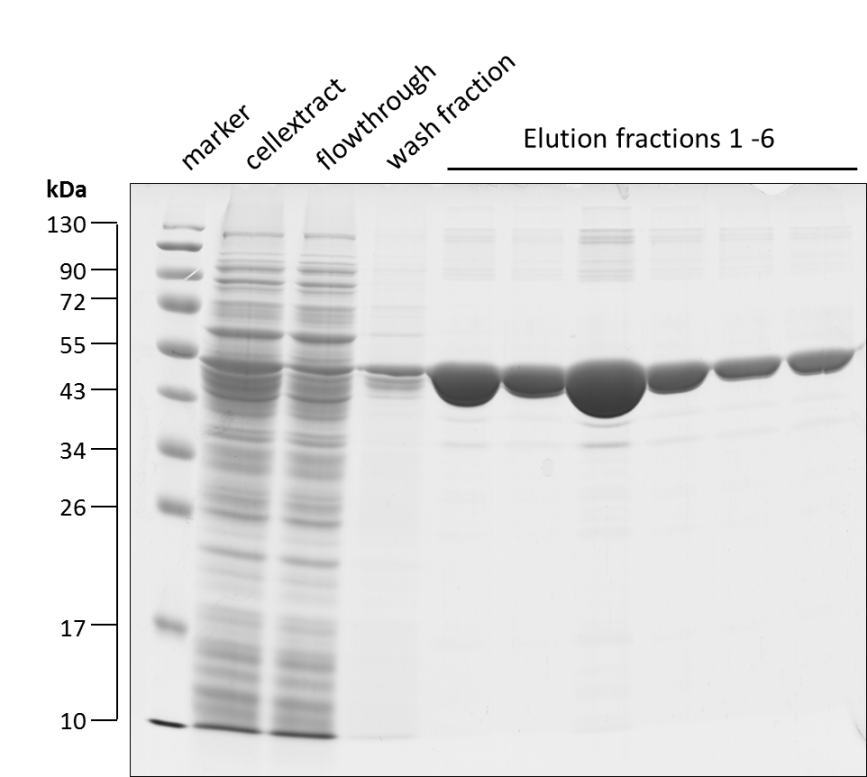
**

**Fig. S1:** Purification of recombinant streptavidin-tagged *C. glutamicum* OtsA from *E. coli* BL21(DE3)(pASK-IBA3-*otsA*): SDS-PAGE analysis of crude cell extract and Streptavidin-affinity chromatography flow-through, wash fraction, and elution fractions. PAGE ruler prestained protein ladder (MBI Fermentas) was used as marker.
